# A machine learning system enables just-in-time risk-stratified sepsis evaluations in the neonatal intensive care unit

**DOI:** 10.1038/s41372-026-02714-w

**Published:** 2026-05-26

**Authors:** Nankee K. Kumar, Arin Phillips, David B. Gootenberg, Zachary A. Vesoulis, Luke T. Viehl

**Affiliations:** 1 Department of Pediatrics, Washington University in St. Louis, St. Louis, MO, USA.; 2 Division of Newborn Medicine, Department of Pediatrics, Washington University in St. Louis, St. Louis, MO, USA.; 3 Division of Infectious Disease, Department of Pediatrics, Washington University in St. Louis, St. Louis, MO, USA.

## Abstract

**INTRODUCTION:**

NICU sepsis evaluation balances rapid antibiotic administration for mortality reduction against unnecessary treatment. Objective risk stratification optimizes resource allocation.

**METHODS:**

We retrospectively studied 191 sepsis evaluations from 136 NICU patients. Sepsis was defined as positive blood culture or clinical sepsis with ≥5 days of antibiotics. A machine learning score (POWS) used cardiorespiratory data 1 h prior to evaluation to quantify sepsis risk. We performed ROC analysis, identified optimal thresholds by Youden Index, and assessed postmenstrual age (PMA) and gestational age (GA) as effect modifiers.

**RESULTS:**

POWS was higher in sepsis versus no sepsis (4.53 vs. 2.14, *p* < 0.005; AUC 0.744 [95% CI: 0.608–0.870]). A POWS threshold of 2.9 provided 71% sensitivity and 81% specificity for detection, stratifying into groups with a sepsis incidence of 4.2% vs. 31.9%. PMA, but not GA, modulated POWS (−0.054 points/week, *p* < 0.0001).

**CONCLUSION:**

POWS discriminates sepsis risk at the time of evaluation.

## INTRODUCTION

Sepsis is a severe, dysregulated immune reaction, most often in response to infection, and is a significant cause of morbidity and mortality in the neonatal intensive care unit (NICU). Sepsis incidence increases with decreasing birth weight and gestational age (GA), and is estimated to occur in 11–32% of NICU hospitalizations for very low birth weight (VLBW) infants, the highest-risk subgroup [1]. Rapid antibiotic treatment is associated with improved outcomes including mortality, making early sepsis identification and treatment a priority [2, 3]. At the same time, clinical signs of sepsis are non-specific and overlap with common clinical manifestations of prematurity, leading to challenges in diagnosis and contributing to unnecessary antibiotic administration [4-6].

Antibiotic exposure in infancy disrupts the formation of healthy gut microbiota and increases the risk of chronic immune and metabolic disease later in life [7]. Antibiotic overuse also contributes to increasing antibiotic resistance. Thus, sepsis evaluation in the NICU requires balancing early treatment against antibiotic overuse.

Objective risk stratification tools may help providers pursue urgent treatment for infants at high risk of sepsis as well as prevent unnecessary antibiotic administration in patients at lower risk. Previous research has demonstrated characteristic vital sign patterns in the preclinical phase of sepsis may predict future deterioration as early as 24 h before clinical recognition [4, 8-13]. These patterns include decreased heart rate (HR) variability, increased number of HR deceleration events, and increased cross correlation of HR and oxygen saturation (SpO_2_) [4, 8-13]. The autonomic dysfunction underlying these patterns likely stems from the systemic inflammatory response to pathogenic bacteria that defines sepsis [10, 14]. Quantitative score-based recognition of these physiologic patterns forms the basis of the heart rate characteristics (HRC) sepsis prediction model, which was associated with a decreased risk of sepsis-related mortality by 22% in a randomized clinical trial of VLBW neonates [15].

A related model, the pulse oximetry warning system (POWS), adds pulse oximetry to the heart rate metrics of HRC and utilizes artificial intelligence (AI) trained to recognize abnormal cardiorespiratory patterns that predict risk of sepsis [11]. This model has been externally validated across three NICUS with acceptable to excellent discrimination (area under the receiver operating characteristic curve [AUC] 0.780–0.808) to identify late onset sepsis 24 h prior to clinical emergence [11].

HRC and POWS were developed and validated using cardiorespiratory data primarily collected from premature infants [4, 10, 11, 13, 15-17]. While these infants have the highest risk of sepsis, most neonatal sepsis evaluations are performed on lower-risk, non-VLBW infants who have a much lower baseline sepsis incidence [18]. As such, most unnecessary antibiotic treatment and clinical resource use occurs in a more mature neonatal population. Although the underlying physiologic phenomena behind POWS and HRC (vagal dysfunction causing abnormal heart rate patterns) should be universal in all infants, it is possible that VLBW infants may have altered or exaggerated heart rate responses due to their immature autonomic nervous and immune systems [19-24]. While not the primary outcome of the study, prior work by Rio et al. supports this hypothesis noting variance in sensitivity and specificity of HRC by GA [25].

### Aims

The NICU sepsis evaluation process involves complex medical decision-making, including the choice of specimens to culture (blood, urine, tracheal aspirate, and/or cerebrospinal fluid), the decision of whether to initiate antibiotics, the choice of antibiotics, and contingency planning when there are limited resources (staffing) or challenges in obtaining specimens. An objective risk score would allow stratification of infants into “high” or “standard” risk groups, where the former may justify bringing maximal clinical resources to the bedside in an urgent manner to obtain cultures and administer antibiotics, while the latter may provide reassurance for a lower intensity management approach as part of an antibiotic and diagnostic stewardship strategy.

In this project, we used sepsis evaluation data collected during an ongoing sepsis quality improvement (QI) project in conjunction with continuous vital sign data to calculate POWS scores. We sought to understand 1) whether POWS accurately predicts sepsis in all NICU patients, across the range of GA and postmenstrual ages (PMA), 2) if “just-in-time” POWS scores, used for risk stratification at the point of sepsis evaluation could augment our current clinical decision making, and 3) whether there is a relationship between time to antibiotic administration and POWS score.

## METHODS

### Patient population and study design

We conducted a single-center retrospective study in the NICU at St. Louis Children’s Hospital (SLCH), a level IV academic NICU which serves urban, suburban, and rural patients in the St. Louis metro area and surrounding parts of Missouri and Illinois. Patients were included if they were admitted to the SLCH NICU and underwent an evaluation for late onset sepsis from November 2024 until September 2025. All sepsis evaluations considered for inclusion were identified through our NICU’s QI initiative to reduce time to antibiotic administration (described below). We excluded sepsis evaluations that occurred as part of a patient’s admission process. We also excluded patients if they were missing vital sign data such that the POWS score could not be calculated.

The study design was reviewed by the Washington University Human Research Protection Office and approved under a waiver of consent. All study procedures were performed in accordance with the relevant local and Federal guidelines and regulations.

### QI initiative to improve time to antibiotics

In November 2024, our NICU began a QI project to reduce time to antibiotic administration. The cornerstone of this initiative was the implementation of a multidisciplinary, structured sepsis huddle at the time of sepsis evaluation: once the clinical team decides to perform a sepsis workup, providers, bedside nurses, and other relevant team members join at the bedside to discuss the clinical indications for the workup, required laboratory tests, and choice of antibiotics. A templated “sepsis huddle” note was entered into the electronic medical record (EMR). Each note includes sepsis huddle start time, which cultures are obtained (e.g., blood, urine, tracheal aspirate, CSF), recent culture results, and which antibiotics are ordered.

### Typical clinical practice

The SLCH sepsis guidelines are based on recommendations from the American Academy of Pediatrics for the workup and treatment of neonatal sepsis [26, 27]. For sepsis evaluation, a complete blood count, basic metabolic labs, urine cultures are simultaneously collected with blood cultures in all patients where there is clinical suspicion of sepsis. Cerebrospinal fluid (CSF) cultures are obtained in infants younger than 28 days with a fever (≥38 °C) and/or infants with neurologic abnormalities such as new onset seizures or unexplained encephalopathy.

Almost all blood cultures include sampling from two sites (one peripheral and one central, or two peripherals if no central line is present). A total volume of 1 mL is drawn from each site and split into two blood culture bottles. Blood cultures are incubated for 4 days on the BACT/ALERT VIRTUO system (bioMerieux SA, Marcy-l'Étoile, France). When a culture has a positive result, gram stain is performed, followed by standard culture and identification on agar plate with susceptibility testing. The cobas ePlex BCID system (Roche Diagnostics, Indianapolis, IN) encompassing rapid multiplex molecular panels with a total of 20 gram-positive targets, 21 gram-negative targets, and 10 bacterial resistance genes, is run alongside the standard culture to more quickly identify bacterial targets and narrow antibiotic coverage.

After cultures are obtained, all patients are started on empiric antibiotics. Infants with suspected late-onset sepsis (LOS, defined as sepsis evaluation after 72 h of life) are treated with intravenous nafcillin and gentamicin. Oxacillin is substituted for nafcillin for patients without a central line, ampicillin is substituted for nafcillin or oxacillin if abdominal pathology is suspected, and ceftazidime is substituted for gentamicin when CSF penetration is needed. Patients with signs of septic shock receive vancomycin and cefepime as an alternative empiric antibiotic regimen to provide broader coverage.

### Clinical data collection

For this project, we reviewed sepsis evaluations identified as part of the previously described QI project and included only those that met all inclusion criteria. For all identified cases, a common dataset was abstracted from the electronic health record (EHR). Data elements included GA at birth (completed weeks), PMA at time of sepsis evaluation, birth weight, sex, race, mortality, hospital length of stay, PMA at discharge, evaluation outcome (culture positive sepsis, clinical sepsis, or no sepsis), other infections (including urinary tract infections, viral infections, and focal infections), and time to antibiotics (elapsed time between huddle start and first antibiotic administration time recorded in the EMR).

We defined “sepsis” inclusively as either a positive blood culture and ≥5 days of antibiotics (“culture-positive sepsis”) or a negative culture with a sepsis-like syndrome treated with ≥5 days of antibiotics (“clinical sepsis”). All other events were classified as “no sepsis,” including sepsis ruled-out and focal infection without bacteremia, for example, pneumonia. These definitions are consistent with previous work on POWS [9-12].

### Continuous vital sign data collection methods

Heart rate data were obtained from the bedside patient monitors (Intellivue MP70, MX800 or MX850, Philips Medical, Andover, MA). SpO_2_ data were obtained using the Nellcor OxiMax pulse oximeter (Medtronic, Boulder, CO) using the neonatal-adult MAX-N or OxySoft adhesive sensors. Time-integrated vital sign data were continuously and prospectively aggregated in a central repository (T3, Etiometry, Boston, MA) at a sampling rate of 0.2 Hz. HR and SpO_2_ data for each included patient were exported retrospectively as CSV files for processing using the POWS algorithm.

### POWS calculations

The Pulse Ox Warning System (or POWS) is a machine learning-derived algorithm that uses histogram-based heart rate and pulse oximetry features to predict future risk of sepsis. Sepsis is marked by decreased HR variability, increased number of deceleration events, and increased cross correlation of HR and SpO_2_ [4, 8-13]. Using the model described by Kausch et al., POWS was calculated using serial, 10-minute, non-overlapping windows of HR and SpO_2_ data [11]. For each window, physiologic features (mean, standard deviation [SD], skewness, kurtosis of both HR and SpO_2_, as well as their minimum and maximum cross-correlation) were calculated. Values for each of these features were then entered into a pre-trained logistic regression model to generate the predicted log-odds for that window, which was converted to a probability between 0 and 1 using an inverse logit function, and finally divided by 0.0026 to generate the relative risk as compared to a daily sepsis risk baseline.

In this study, individual POWS score components (standard deviation, skewness, kurtosis, and cross correlation of SpO_2_ and HR) were calculated using 10-minute windows of raw physiological data sampled at 0.2 Hz, an identical window length but slightly lower sampling rate compared to 0.5 Hz in the validation study [11]. The final composite POWS value used in this study represents the average of the six 10-min windows in the 60 min prior to the recorded sepsis huddle time, rather than the 4-h smoothed and recalibrated values used in the original POWS implementation. This difference reflects very different use cases; a continuous predictive risk model differentiating few sepsis events over long hospitalization periods (original study) vs. point-of-care adjunctive risk stratification where there is already clinical suspicion for sepsis. Consequently, the discrimination thresholds determined in this study are specific to this use case.

### Statistical analysis

We used descriptive statistics to assess clinical characteristics of the cohort and stratified analyses by PMA category (<32, 32–35, ≥36 weeks). We conducted pairwise comparison tests (Wilcoxon rank sum test and Kruskal-Wallis test) to analyze relationships between time to antibiotics and sepsis outcome, POWS scores and sepsis outcome, and time to antibiotics and POWS risk categories. We performed receiver operating characteristic analysis to assess POWS predictive ability with 95% confidence intervals estimated using stratified bootstrap resampling (2000 iterations). To identify optimal cutoffs for sepsis risk stratification, we bootstrapped the Youden index (2000 iterations with stratified sampling) to obtain the median optimal threshold and 95% confidence intervals.

To evaluate whether PMA modifies POWS predictive ability, we compared four logistic regression models: POWS only, PMA only, POWS + PMA (additive), and POWS × PMA (interaction), selecting the best model by Akaike Information Criterion (AIC). We used likelihood ratio tests to compare nested models and assess whether PMA interaction terms significantly improved model fit. We additionally evaluated whether gestational age at birth modified POWS performance using similar model comparisons. We assessed PMA modulation of POWS scores using Spearman correlation and linear regression. Sensitivity, specificity, positive predictive value (PPV), and negative predictive value (NPV) were calculated at candidate POWS thresholds and stratified by PMA group. Statistical significance was defined as *p* < 0.05.

Positive and negative likelihood ratios (LR+ and LR−) were calculated at candidate thresholds to assess clinical utility. We evaluated whether PMA and birth GA modified the relationship between sepsis outcome and time to antibiotic administration using linear regression models with interaction terms, comparing nested models by likelihood ratio tests. Time to antibiotics was calculated as the interval between sepsis huddle and first antibiotic administration.

All analysis was performed using R version 4.5.2 (The R Foundation for Statistical Computing, Vienna, Austria) using the *ggplot2, pROC,* and *tidyverse* packages.

## RESULTS

### Characteristics of sepsis evaluations

191 sepsis evaluations from 136 patients met inclusion criteria. The mean birth GA was 30 ± 5 weeks, and the mean birth weight was 1646 ± 950 g. While the median PMA at the time of sepsis evaluation was 35.6 weeks (IQR 30.4–40.9), most workups occurred while infants were still quite immature or at term or near-term age, with 66, 32, and 93 evaluations being performed at <32 weeks, 32–35 weeks and ≥36 weeks PMA, respectively (Supplemental Table S1). No sepsis occurred in 170/191 (89%) of evaluations and was the most common outcome, while sepsis (including both culture-positive sepsis and clinical sepsis) occurred in 21/191 (11%) evaluations. Comparing sepsis to no sepsis (Table 1), patients with sepsis had lower PMA at evaluation (*p* = 0.004), higher overall mortality (*p* = 0.004), and a trend toward earlier GA (*p* = 0.10).

Within the culture positive sepsis group, methicillin-susceptible *Staphylococcus aureus* was the most common causative organism, identified in 4/14 (29%) of cases (Table 2). Among patients who had focal infections, urinary tract infection was the most common, followed by tracheitis. The most common organism identified in urinary cultures was *E. coli,* identified in 5/16 (31%) of cases.

### Model performance

**Fig. 1:**
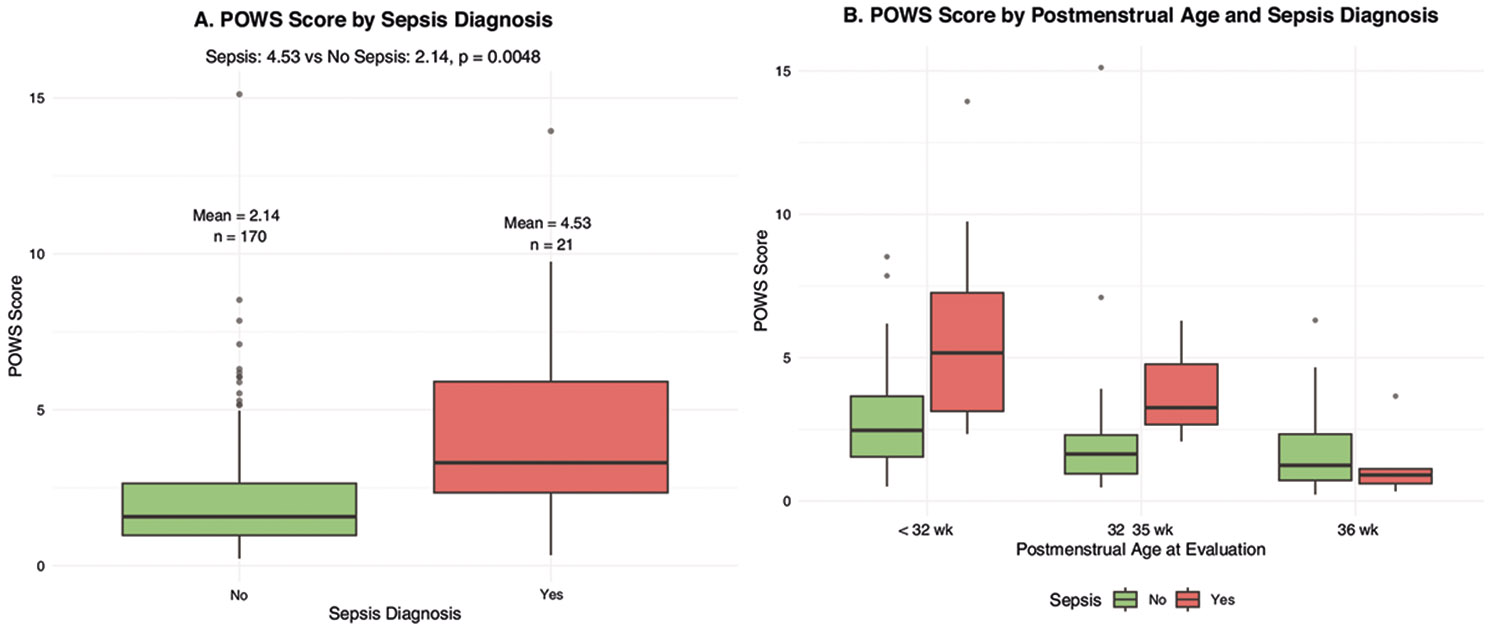
Stratified POWS scores. Box and whisker plots of POWS score for sepsis outcomes are shown in **A**, with a significantly higher POWS score for those with a sepsis diagnosis. When stratifying by PMA at time of evaluation (**B**), POWS scores decrease overall as PMA increases while the difference between sepsis and no sepsis also decreases.

Mean POWS scores were significantly higher in sepsis than no sepsis, with more than a two-fold difference between groups (4.53 vs. 2.14, *p* = 0.005, Fig. 1A). In ROC analysis, the AUC was 0.744 (95% CI 0.608–0.870) for the detection of sepsis. Model (95% CI 2.07–3.28), which we rounded to 2.9 for clinical application (Supplementary Fig. S3). This threshold provided excellent stratification for sepsis risk, with an incidence of sepsis three times greater than the baseline incidence assumption (30.6% vs. 10%) in the “high risk” score group (≥2.9) and less than half the baseline incidence assumption (4.2% vs. 10%) in the “standard risk” score group (<2.9, Supplementary Fig. S4).

### Risk modification with advancing PMA at evaluation

**Fig. 2:**
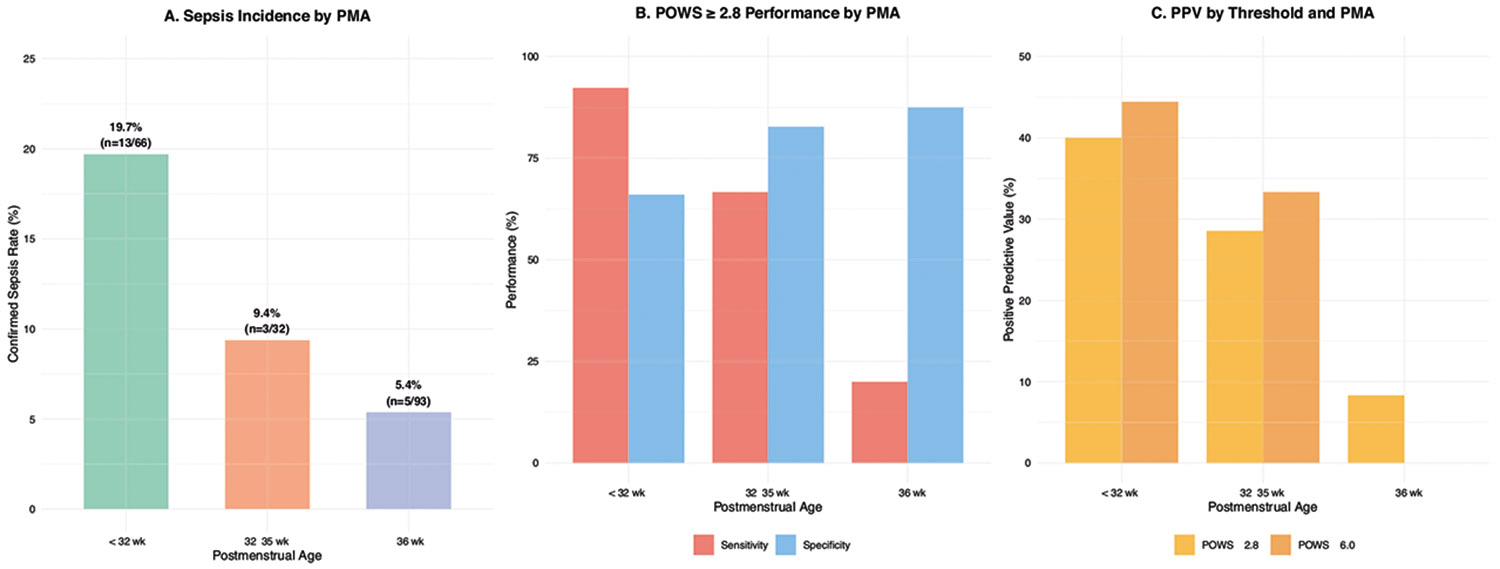
Sepsis incidence and POWS performance by PMA. As would be expected, sepsis incidence decreases with increasing PMA at time of evaluation (**A**). This shift in incidence changes the predictive performance of POWS, trading sensitivity for specificity with increasing PMA (**B**). The positive predictive value (PPV) of POWS is greatest for PMA < 32 weeks (**C**).

As would be expected, the prevalence of sepsis among evaluated infants decreased with increasing PMA, falling from 19.7% for evaluations performed on infants <32 weeks PMA to only 5.4% for infants ≥36 weeks PMA at the time of evaluation (Fig. 2A). While sepsis generates higher POWS compared to no sepsis, increasing PMA was significantly and independently associated with lower POWS scores (Spearman *ρ* = −0.433, *p* < 0.0001), with a decrease of approximately 0.054 points per week of advancing PMA (Fig. 1B).

The changing prevalence of sepsis and maturational changes in POWS scores that occurs with increasing PMA has important implications for algorithm performance. We examined whether PMA at sepsis evaluation modifies POWS predictive ability using logistic regression models. Four models were compared: POWS-only, PMA-only, POWS + PMA (additive), and POWS × PMA (interaction). The POWS × PMA interaction model demonstrated the best fit (AIC 115.77), superior to the POWS-only model (AIC 120.63), suggesting that PMA does modify the relationship between POWS and sepsis risk. POWS had a decreasing predictive effect across PMA strata, with mean POWS differences between sepsis and no sepsis cases of 3.00 points in infants <32 weeks, 1.57 points in infants 32–35 weeks, and −0.28 points in infants ≥36 weeks (Fig. 1B). Using the POWS threshold of ≥2.9, the peak sensitivity was 92.3% for evaluations performed <32 weeks PMA but fell to only 20.0% by term/near-term PMAs. Specificity increased from 66.0% to 87.5% over the same strata (Fig. 2B).

In contrast, birth gestational age exerted limited influence on POWS’ predictive performance. When we compared logistic discrimination was higher at lower PMAs compared to the highest PMA group (Supplementary Fig. S2). A 2000 iteration bootstrap estimation of the Youden Index revealed that sensitivity and specificity were maximized at median POWS threshold of 2.88 regression models using birth GA instead of PMA—POWS-only, GA-only, POWS + GA (additive), and POWS × GA (interaction)—the POWS-only model remained the best fit (AIC = 120.6), superior to POWS + GA (AIC = 122.6) and POWS × GA (AIC = 121.2). The likelihood ratio test indicated that birth GA did not significantly modify POWS risk prediction (*p* = 0.064).

### POWS threshold comparison

We evaluated two candidate thresholds for clinical implementation: a Youden-optimal threshold of 2.9 derived from bootstrapped analysis of our cohort (median 2.88, 95% CI: 2.07–3.28, Supplementary Fig. S3), and a literature-based threshold of 6.0 from prior POWS validation studies [16]. Using the 2.9 threshold, 47 episodes were classified as “high risk” and 15/47 (31.9%) of them returned a true sepsis diagnosis. In contrast, the other 144 episodes classified as “standard risk” had a true positive sepsis diagnosis returned in only 6/144 cases (4.2%) which is less than half the assumed sepsis incidence on a clinical basis alone. Thus, the 2.9 threshold demonstrated sensitivity of 71.4%, specificity of 81.2%, PPV of 31.9%, and NPV of 95.8%. The positive likelihood ratio (LR+) of 3.54 indicates that infants with POWS ≥ 2.9 are approximately 3.5 times more likely to have sepsis, while the negative likelihood ratio (LR−) of 0.41 suggests that those below threshold have substantially reduced sepsis probability. In contrast, the higher 6.0 threshold classified 13 patients as “high risk” and 5/13 (38.5%) returned a true sepsis diagnosis. This is compared to a true sepsis rate of 8.7% in those classified as “standard risk” patients (Supplementary Fig. S4). While this threshold achieved higher specificity (95.3% vs. 81.2%) and stronger LR+ (5.06), it sacrificed sensitivity (23.8% vs. 71.4%) and resulted in a higher LR− (0.80 vs. 0.41), meaning that negative tests provide less reassurance. Critically, the 2.9 threshold generates a “standard risk” group with a low rate of sepsis (4.2%), below the assumed baseline NICU sepsis prevalence of 10%. When using a threshold of 6.0, the resulting “standard risk” group has a sepsis rate (8.7%) similar to the assumed baseline range, which limits clinical utility in the context of resource allocation. These performance characteristics suggest that the 2.9 threshold better serves the dual clinical goals of identifying elevated risk infants who warrant aggressive empiric management and a truly low-risk group suitable for standard of care management.

Both thresholds of POWS demonstrate diminishing clinical utility with increasing PMA in our study population. At ≥36 weeks, the POWS ≥ 6.0 threshold predicts only 1 of 93 evaluations to be true sepsis and was incorrect in that prediction (PPV 0%). In contrast, the POWS ≥ 2.9 threshold predicts 12/93 of term/near-term evaluations to be sepsis, resulting in a PPV of 8.3%. Taken together, these findings suggest there is no universal POWS threshold for sepsis risk stratification across PMA (Fig. 2C).

### Sepsis treatment speed

Median time to antibiotic administration, measured from time of sepsis huddle, was 61 min (IQR 46–88 min). The median time to antibiotics was 33% longer in patients with sepsis than in those without (80 vs. 60 min, *p* = 0.03). There was no relationship between POWS score prior to sepsis evaluation and time to antibiotic administration (Spearman *ρ* = −0.032, *p* < 0.658), Supplementary Fig. S5).

## DISCUSSION

Our study evaluated whether POWS, a sepsis risk score, could stratify sepsis risk for NICU patients where there was already clinical concern for infection. Sepsis occurred in 11% of 191 evaluations; two-thirds were sepsis ruled-out and one-quarter were focal infections without bacteremia. POWS significantly predicted sepsis (AUC of 0.744, *p* < 0.001). At the optimal threshold of 2.9 (Youden Index), POWS provided 71% sensitivity and 81% specificity with an excellent negative predictive value of 96%. A POWS threshold of 2.9 provided good risk stratification, with a 4.2% incidence of sepsis below threshold and a 31.9% incidence above (a 7.6-fold difference). Critically, PMA at the time of evaluation significantly modulated POWS scores. Birth GA, by contrast, did not significantly modify POWS effect.

The 11% sepsis prevalence in our cohort underscores the challenge of clinical decision-making when there is concern for infection: even when sepsis is strongly suspected, most infants do not have sepsis. Paradoxically, time to antibiotics was 33% longer in sepsis cases, indicating a discordance between risk and task performance. POWS, which reveals subclinical physiology resulting from evolving sepsis, does not correlate with time to antibiotics. This discrepancy likely reflects the multifactorial nature of treatment timing, including technical challenges with vascular access, need for stabilization interventions, and provider workflow factors (i.e., patient acuity), rather than diagnostic certainty. Younger and more critically ill neonates, who are more likely to have sepsis, also have more technically challenging septic workups. Our findings suggest that POWS used at the time of evaluation may help risk-stratify which evaluations will yield positive cultures and require treatment, independent of clinical logistics.

A sepsis evaluation is always a major event that involves multiple painful procedures for the infant and a long sequence of tasks that must be performed without error by providers to avoid contamination and complications. The burden on patients and providers is heightened in the highest complexity patients. As such, high POWS scores may encourage reluctant providers to move forward with a challenging septic workup based on a more accurate understand of risk and benefits. It can also be used to justify calling for additional resources (e.g., extra nurses, providers with greater experience at venipuncture, respiratory therapy) at the outset. While both measures may decrease time to antibiotics for the sickest patients, the ultimate goal is equity between populations, where technology enables a similar time to antibiotics, regardless of underlying patient characteristics.

The AUC reported here differs from prior POWS validation studies in several important ways. First, we assessed POWS discrimination at the time of clinical sepsis evaluation rather than across all patient-hours, representing a more difficult classification task with likely greater clinical impact (distinguishing concerning presentations with sepsis versus without sepsis, rather than healthy infants from sepsis). Second, our outcome included both culture-positive and clinical sepsis cases, whereas prior validation used culture-positive sepsis only. Third, our case mix was limited to infants where clinicians had sufficient concern to initiate formal sepsis evaluation. These methodological differences preclude direct comparison of AUC values between studies, and our results should be interpreted as discrimination performance at the point of clinical decision-making rather than as a continuous monitoring tool.

Prior work established that machine learning algorithms can predict neonatal sepsis, predominantly in high-risk VLBW cohorts with reported AUCs of 0.780-0.808 [4, 10-13, 16]. Our study addresses a fundamentally different and more challenging classification problem: discriminating true from false sepsis alarms among infants where clinical suspicion was already sufficient to trigger formal evaluation. The 11% sepsis rate in our cohort represents a 42-fold enrichment over the baseline daily sepsis risk in unselected NICU populations (0.26% in the POWS training dataset), indicating that our entire cohort was already at significantly greater risk for sepsis than the general NICU population. In this context, our AUC of 0.744 demonstrates that POWS further stratifies risk even within this pre-selected population—a considerably harder task than discriminating healthy infants from those with sepsis.

An important novel finding is that PMA at evaluation significantly modulates POWS scores and may modify sepsis risk prediction. While birth gestational age did not significantly interact with POWS, the PMA-POWS relationship suggests that developmental maturity at assessment, not historical prematurity, governs physiologic response patterns. We developed a model incorporating PMA alongside POWS and found that it offered better discrimination in our dataset than POWS alone. This maturational effect is only compounded by the decreasing prevalence of sepsis which occurs over the same time period (a 4-fold decrease observed in this study). These findings warrant investigation into PMA-adjusted POWS thresholds for future implementation.

Our study uniquely evaluated POWS as a real-time, “point-of-care” decision support tool. The 2013 Fairchild trial demonstrated that bedside display of HRCs reduced 30-day sepsis mortality in VLBW patients, suggesting mortality benefit from timely risk assessment [17]. However, this study did not define the time horizon or score threshold for incorporation into clinical decision making. Our study builds on this research and suggests that adding “just-in-time” POWS score on top of clinical suspicion may optimize the value of each of these strategies by maximizing sensitivity and specificity while reducing the risk of false alarms. Our findings suggest that objective risk stratification may help decouple decisions regarding treatment urgency from the technical and resource constraints that currently influence antibiotic timing. In the clinical setting, POWS is meant to serve as a singular data point, among many other data points such as laboratory tests and clinical exam findings, which inform providers’ decision making. Although its discrimination between high and standard risk groups is meaningful, POWS is not meant to dissuade clinicians from performing septic workups or administering antibiotics when there is clinical concern. Future refinements may enable the development of an additional risk tier where laboratory workup *without* antibiotics is the appropriate management, but current data do not yet support such a threshold.

Important limitations qualify our findings. Selection bias is inherent: all patients had clinical concern for sepsis, thus, evaluated POWS performance is specific to symptomatic cohorts and not reflective of asymptomatic populations. Our single-center cohort of 191 evaluations from 136 patients may be under-powered to detect PMA-specific interactions. This single-center study assessed POWS performance in a specific implementation context (sepsis huddle workflow) that may not generalize to other institutions. Multicenter validation would strengthen these findings and assess performance across different patient populations and clinical workflows. The definition of “sepsis-like syndrome” with ≥5 days of antibiotics captures cases of prolonged empiric coverage despite negative cultures, may also represent severe illness of non-sepsis origin. In this study, the Youden index identified a mathematically optimal threshold which maximized sensitivity and specificity in this dataset. However, clinical implementation would require consideration of additional factors including resource utilization, antibiotic stewardship goals, and institutional/provider risk tolerance. These thresholds should not be applied clinically without prospective validation. Finally, as POWS was calculated retrospectively, we are unable to identify the impact of score availability on provider behavior or patient outcomes. These limitations underscore the need for prospective, multi-center validation before widespread implementation.

## CONCLUSION

We conducted a single-center retrospective study to assess whether POWS, a machine learned sepsis risk score, trained on heart rate and oxygen saturation patterns, could risk-stratify NICU patients at the time of sepsis evaluation. POWS provided excellent discrimination between sepsis and no-sepsis cases, as sepsis cases had 2.1-fold higher scores (4.53 vs. 2.14, *p* < 0.001) with AUC = 0.744. A POWS threshold of 2.9 identified clinically actionable groups—standard risk (4.2% sepsis rate, below NICU baseline) and high risk (31.9% sepsis rate, above baseline) warranting urgent escalation. A novel finding was that postmenstrual age at evaluation significantly modulates both POWS magnitude and predictive performance, whereas birth gestational age does not. This distinction suggests POWS interpretation should be calibrated to current developmental maturity and expected sepsis incidence rather than birth demographics.

The 11% sepsis incidence we identified in our population, along with a longer time to antibiotics in true sepsis cases (80 vs. 60 min), demonstrates that even if providers have clinical suspicion of sepsis, technical and logistical difficulties may prevent them from early antibiotic administration. By leveraging ML to provide clinicians with additional data, prospective implementation of real-time POWS display may enable objective risk-informed triage, encouraging providers to bring maximal resources to bedside at the initiation of a septic workup in high-risk infants, while supporting antibiotic stewardship in lower-risk cases.

## Supplementary Material

Supplemental Material

**Supplementary information** The online version contains supplementary material available at https://doi.org/10.1038/s41372-026-02714-w.

## Figures and Tables

**Table 1.** Patient characteristics.

Characteristic	No Sepsis *N* = 170	Sepsis *N* = 21	*P*-value
Gestational age (weeks), median (IQR)	29.0 (26.0, 34.0)	26.0 (25.0, 31.0)	0.10
PMA at sepsis evaluation (weeks), median (IQR)	36 (31, 41)	30 (28, 34)	**0.004**
Birth weight (grams), median (IQR)	1270 (853, 2005)	910 (760, 1480)	0.3
Female sex, *n* (%)	87 / 170 (51%)	9 / 21 (43%)	0.5
Race, *n* (%)			
Asian	1 / 170 (0.6%)	0 / 21 (0%)	0.5
Black or African American	39 / 170 (23%)	8 / 21 (38%)	
Multiple Race	8 / 170 (4.7%)	0 / 21 (0%)	
Native Hawaiian or Other Pacific Islander	2 / 170 (1.1%)	0 / 21 (0%)	
White	119 / 170 (70%)	13 / 21 (62%)	
Hispanic ethnicity, *n* (%)	6 / 170 (3.5%)	0 / 21 (0%)	>0.9
Mortality	26 / 170 (15%)	9 / 21 (43%)	**0.004**
Length of initial hospitalization (days), median (IQR)	108 (63, 193)	154 (32, 211)	0.7
PMA at discharge (weeks), median (IQR)	45 (40, 58)	51 (33, 59)	0.8

*IQR* interquartile range, *PMA* postmenstrual age.

Bold values indicate statistical significance at p < 0.05.

**Table 2.** Sepsis evaluation outcomes.

Sepsis category	*n* (%)
Sepsis	**21 (11)**
Clinical sepsis without positive blood culture	7 (3.6)
Blood culture-positive sepsis	14 (7.1)
S. aureus (methicillin sensitive)	4 (2.0)
Coagulase-negative staphylococcus	2 (1.0)
Klebsiella pneumoniae	2 (1.0)
Enterococcus faecalis	2 (1.0)
Other organisms	4 (2.0)
No sepsis	**170 (89)**
Sepsis Ruled-out	120 (70.5)
Focal Infection	50 (29.4)
Tracheitis	12 (7.1)
E. coli	1 (0.5)
MRSA	1 (0.5)
Other organisms	10 (5.8)
Urinary tract infection	16 (9.4)
E. coli	5 (2.9)
K. pneumoniae	1 (0.5)
Other organisms	10 (5.8)
Pneumonia	4 (2.4)
Cellulitis	5 (2.9)
Meningitis	2 (1.2)
Necrotizing enterocolitis	4 (2.4)
Peritonitis	1 (0.5)
Other focal infections	4 (2.4)
**Total**	**191 (100)**

*MRSA* methicillin Staphylococcus aureus.

Bold values indicate statistical significance at p < 0.05.

## Data Availability

Source code for the underlying analysis is freely available at https://github.com/Vesoulis-Lab. Patient level data cannot be disclosed due to privacy restrictions.

## IMPACT:

- A machine learning-developed score (POWS) provides real-time risk stratification during sepsis evaluations, distinguishing a truly low-risk group (4.2% sepsis rate) from a high-risk group (31.9% sepsis rate), among infants with clinical concern for infection.
- Postmenstrual age at evaluation significantly modulates POWS’s performance but birth gestational age does not, suggesting that current developmental maturity is more important than historical prematurity when interpreting and utilizing POWS.
- Prospective implementation with real-time POWS display may enable risk-based triage thereby prioritizing resources to pursue urgent workup and early antibiotic administration in high-risk infants.
