# Supplementary material for: A machine learning system enables just-in-time risk-stratified sepsis evaluations in the neonatal intensive care unit
Source: J Perinatol. Author manuscript; Available in PMC 2026 Jun 21. (PMC13283304; doi:10.1038/s41372-026-02714-w)
Supplement: Supplemental Material [file NIHMS2182349-supplement-Supplemental_Material.docx]

**Supplementary Materials - A Machine Learning System Enables Just-In-Time Risk-Stratified Sepsis Evaluations in the Neonatal Intensive Care Unit**

**Supplemental Table S1**: Sepsis evaluations and incidence by PMA group

| **PMA group** | **N** | **Sepsis diagnosis** | **Sepsis incidence (%)** | **Median age (days)** |
| --- | --- | --- | --- | --- |
| < 32 weeks | 66 | 13 | 19.7% | 13.0 |
| 32-35 weeks | 32 | 3 | 9.4% | 35.5 |
| ≥ 36 weeks | 93 | 5 | 5.4% | 64.0 |

**Supplemental Figure S2**: ROC curves for the total cohort and by PMA subgroups

**
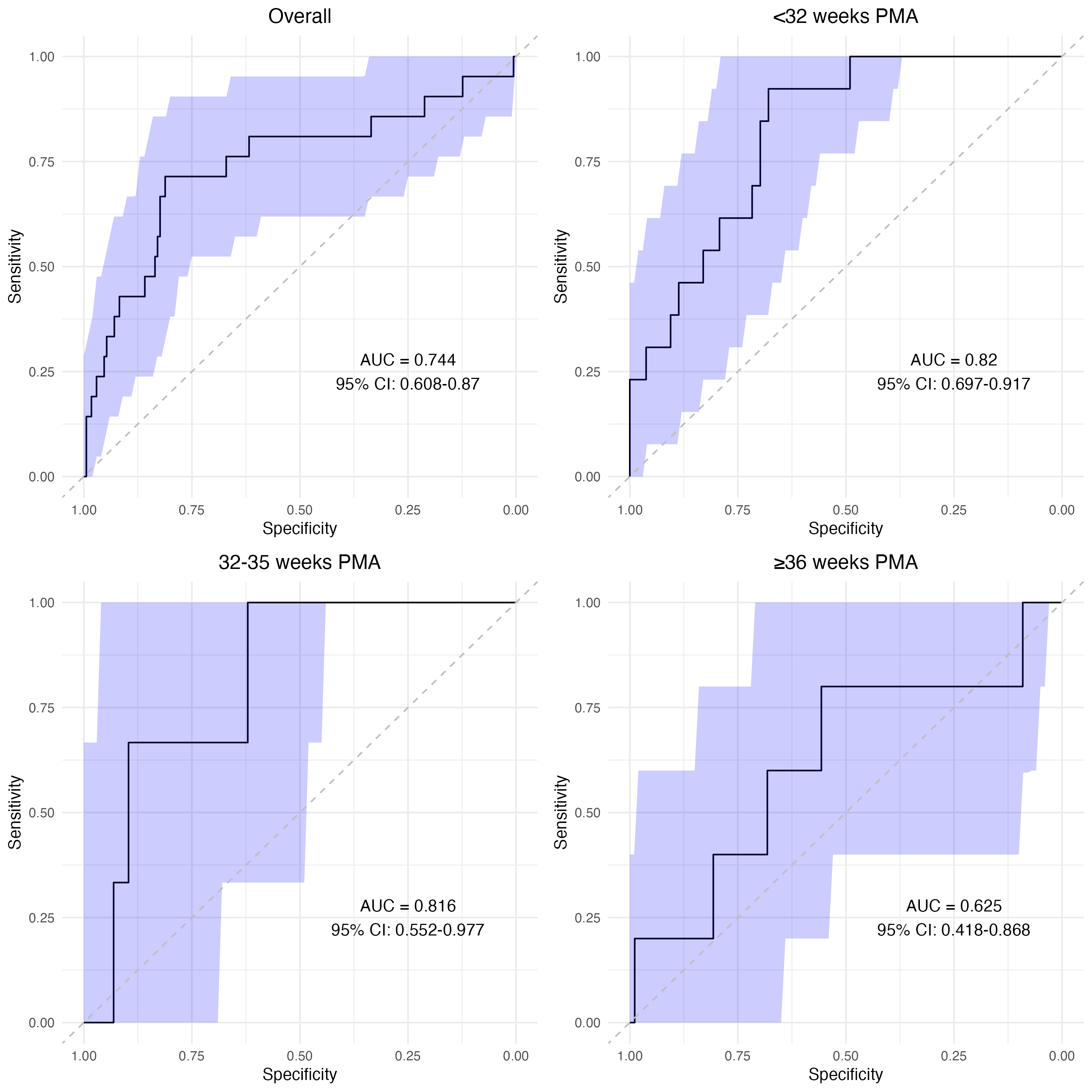
**

**Caption:** Plot of the area under the ROC curves (AUC) for the overall cohort (upper left) and each of the three PMA subgroups are shown. Bootstrapping with 2000 iterations was used to identify the 95% CI, which is shaded in purple. Note the decreased performance in the higher PMA group (bottom right) influenced by a low incidence of sepsis.

**Supplemental Figure S3:** Histogram of POWS threshold identified by bootstrapping

**
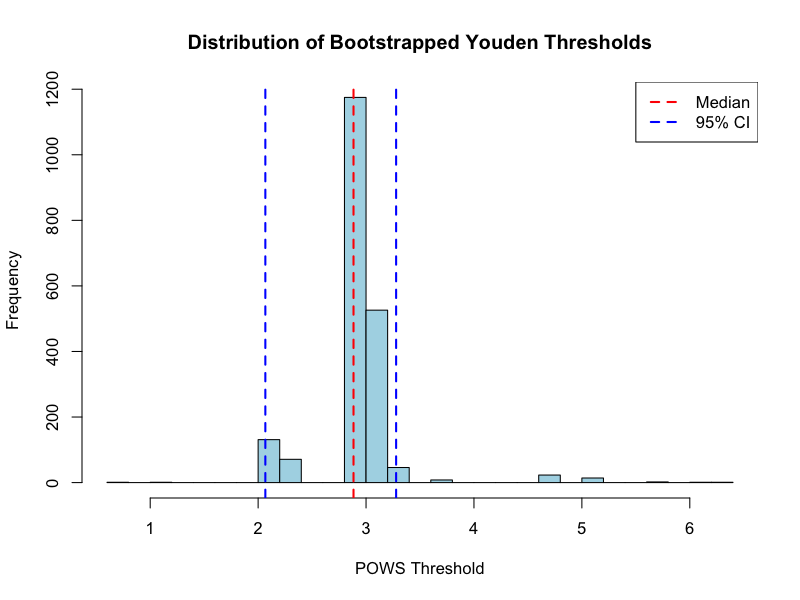
**

**Caption:** The optimal POWS threshold was determined by a 2000 iteration bootstrapping of the Youden index. Shown is a histogram of the results where a median value of 2.88 was identified. Estimates were heavily clustered around 3.

**Supplemental Figure S4:** Sepsis incidence by POWS risk group and threshold


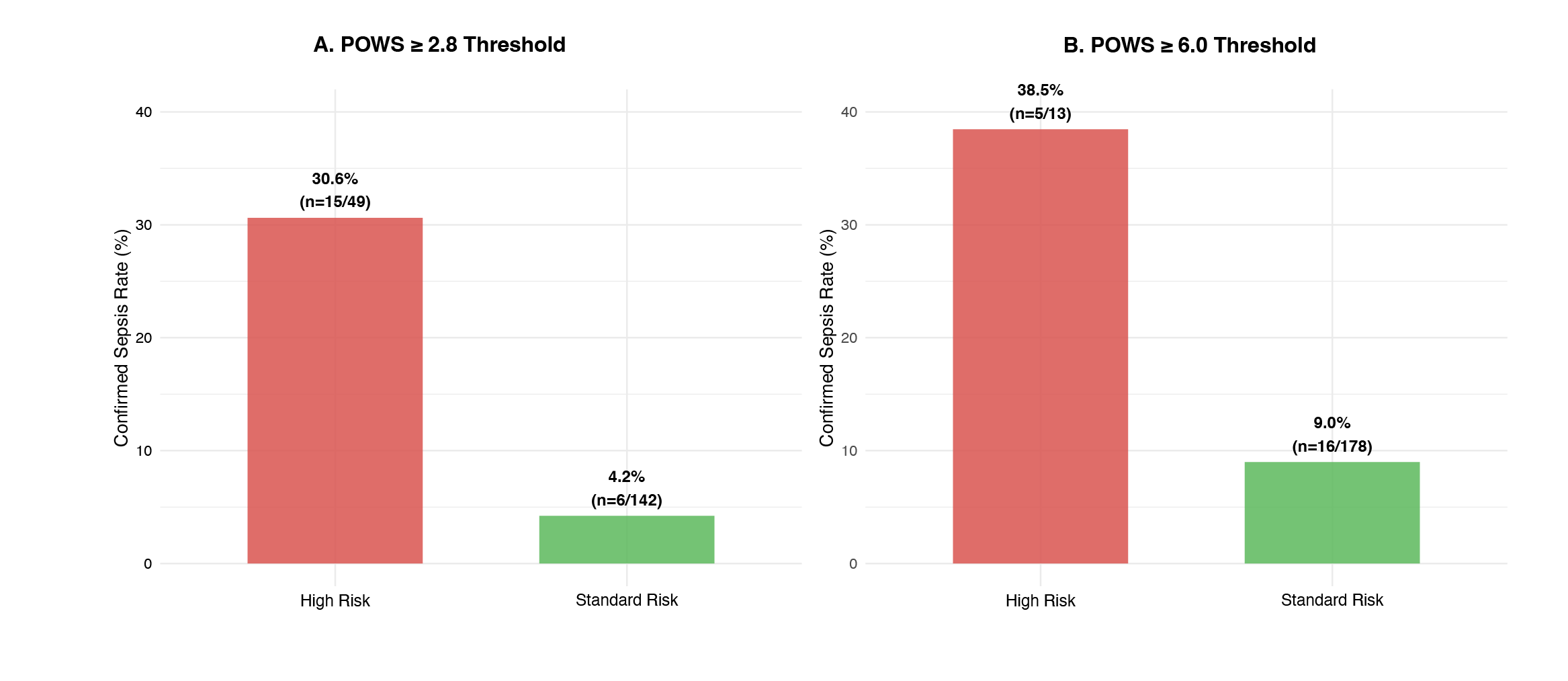


**Caption:** Sepsis incidence by risk group (above or below threshold) is shown for thresholds of 2.9 and 6.0. While both thresholds provide identification of enriched sepsis risk groups, the threshold of 2.9 differentiates a “high risk” group from that “standard risk” group with an incidence below the baseline estimated risk of 10%. In contrast, the “standard risk” group when using the 6.0 threshold is very similar to the baseline assumption.

**Supplemental Figure S5**: Time to antibiotics and POWS


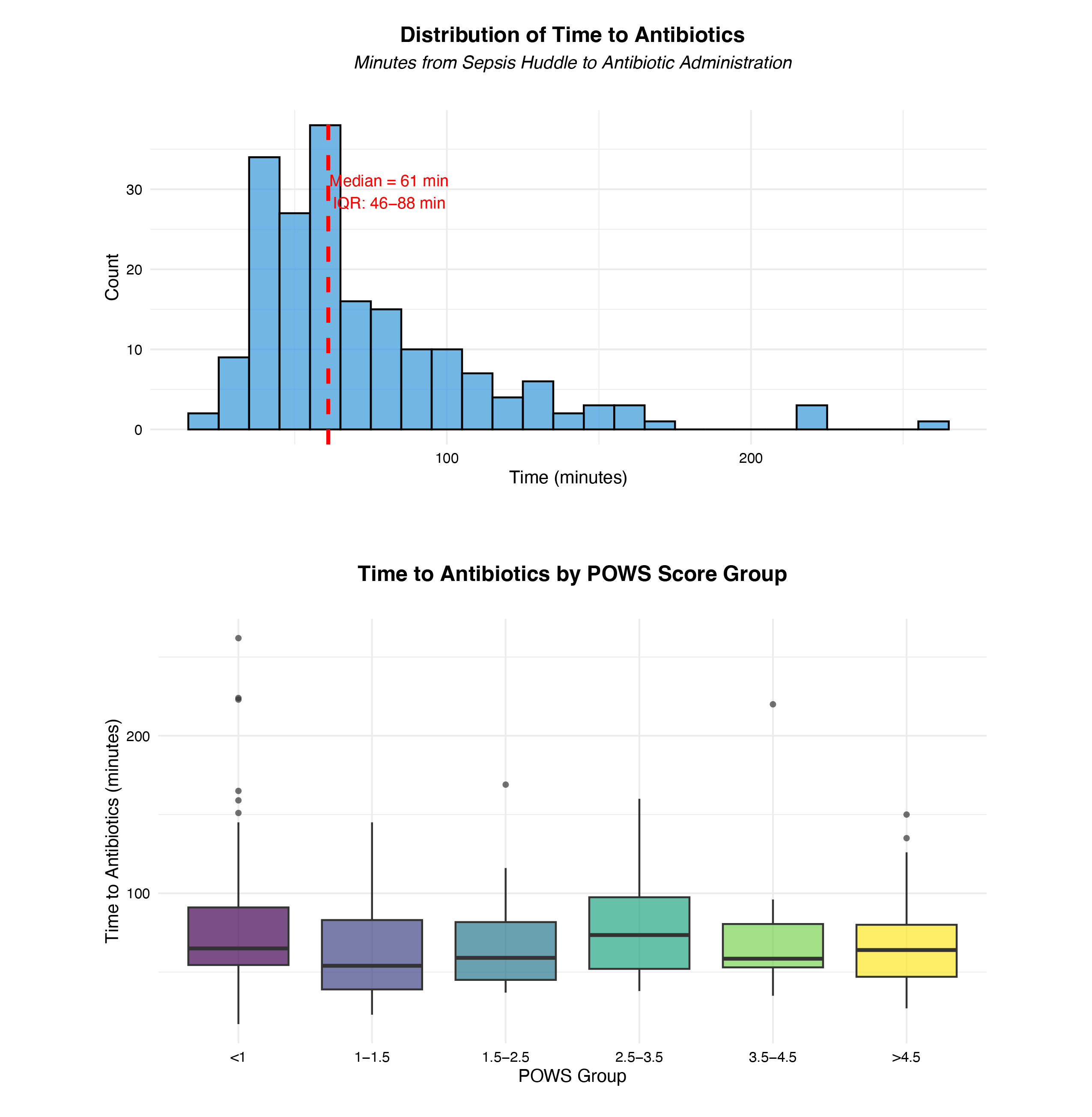


Caption: The median time to antibiotic administration after the sepsis huddle was 61 minutes with an IQR of 46-88 minutes (top panel). Infants with sepsis had a longer median time to antibiotics (80 vs 60 min, p-0.03). Time to antibiotics was not related to the POWS score in the hour preceding the huddle (bottom panel).
